# Inflammatory biomarkers in patients with sciatica: a systematic review

**DOI:** 10.1186/s12891-019-2541-0

**Published:** 2019-04-09

**Authors:** Maarten J. Jungen, Bastiaan C. ter Meulen, Tim van Osch, Henry C. Weinstein, Raymond W. J. G. Ostelo

**Affiliations:** 1 Department of Neurology, OLVG, Amsterdam, The Netherlands; 2 Department of Neurology, Zaans Medisch Centrum, Zaandam, The Netherlands; 3 Department of Health Sciences, VU University, De Boelelaan, 1081 Amsterdam, HV Netherlands; 4 Department of Epidemiology and Biostatistics and the Amsterdam Movement Sciences Research Institute, Amsterdam UMC, De Boelelaan, 1081 Amsterdam, HV Netherlands

**Keywords:** Systematic review, Sciatica, Lumbar disc herniation, Inflammation, Biomarkers, Cytokines, Interleukin

## Abstract

**Background:**

This systematic review focusses on inflammation as an underlying pathogenic mechanism in sciatica. We addressed two questions in particular: (1) what inflammatory biomarkers have been identified in patients with sciatica in the literature so far? 2) is there an association between the level of inflammatory activity and clinical symptoms?

**Methods:**

The search was conducted up to December 19th 2018 in MEDLINE, EMBASE, CENTRAL and Web of Science. The study selection criteria: (1) observational cohort studies, cross-sectional studies and randomized clinical trials (RCT), (2) adult population (≥ 18 years) population with sciatica, (3) concentrations of inflammatory biomarkers measured in serum, cerebrospinal fluid (CSF) or biopsies, and (4) evaluation of clinically relevant outcome measures (pain or functional status). Three reviewers independently selected studies and extracted data regarding the study characteristics and the outcomes. Risk of Bias was evaluated using an adjusted version of the Quality in Prognosis Studies (QUIPS) tool.

**Results:**

In total 16 articles fulfilled the criteria for inclusion: 7 cross sectional observational studies and 9 prospective cohort studies that included a total of 1212 patients. With regard to question 1) the following markers were identified: interleukin (IL)-1β, IL-2, IL-4, IL-6, IL-8, IL-10, IL-17, IL-21, tumor necrosis factor-α (TNF-α), phospholipase A2, high sensitivity C-reactive protein (hsCRP), C-X-C motif chemokine 5 (CXCM5), CX3CL1, CCL2, epidermal growth factor (EGF), and monocyte chemotactic protein 4 (MCP-4). With regard to question 2) several positive correlations were found in longitudinal studies: a strong positive correlation between inflammatory mediators or byproducts and pain (measured by visual analogue scale, VAS) was found for IL-21 in two studies (r > 0,8), and moderate positive correlations for TNF-a in both serum (r = 0,629) and biopsy (r = 0.65); severe pain (VAS > 4) is associated with increased hsCRP levels among patients with sciatica (adjusted OR = 3.4 (95% CI, 1.1 to 10).

**Conclusion:**

In this systematic review there was considerable heterogeneity in the type of biomarkers and in the clinical measurements in the included studies. Taking into account the overall risk of bias of the included studies there is insufficient evidence to draw firm conclusions regarding the relationship between inflammation and clinical symptoms in patients with sciatica.

**Electronic supplementary material:**

The online version of this article (10.1186/s12891-019-2541-0) contains supplementary material, which is available to authorized users.

## Background

Sciatica or lumbosacral radicular syndrome is characterized by pain radiating into the leg along the course of one of the lumbar nerve roots [1]. Sometimes there is numbness or tingling in the dermatomal distribution of a nerve root. Paresis is present almost half of patients, for example weakness of plantar flexion in S1 radiculopathy. Most patients experience back pain also. The incidence of sciatica in The Netherlands is 9.4 cases per 1000 adults per year [2]. Sciatica is a major cause of costs of hospital care and costs resulting from absenteeism from work [3].

Sciatica is considered having different pathogenic components. First, there is a mechanic component that consists of compression of the nerve root by a herniated disc. Neuroradiologic studies confirm that approximately 90% of cases of sciatica are associated with a disc disorder [4, 5]. Second, it has been hypothesized that inflammation may play a role in patients with low back pain [6] and sciatica [7], the elderly in particular [8] A range of pro- and anti-inflammatory proteins has been found in serum, CSF and biopsies of patients with sciatica, including interleukin (IL)-1β, IL-6, IL-8 and tumor necrosis factor (TNF)-α [7]. Third, in patients with sciatica there possibly is also a neuropathic component caused by neural damage at the level of the nerve root [9].

In this systematic review we focus on the role that inflammation may play in lumbosacral radicular syndrome. We conducted this review as an inflammatory substrate in patients with sciatica could be a potential target for anti-inflammatory therapy, specifically non-steroidal anti-inflammatory drugs (NSAIDs) or transforaminal epidural corticosteroids. We address two questions in particular: (1) what inflammatory biomarkers have been identified in patients with sciatica 2) Is there an association between the level of inflammatory activity and clinical symptoms?

## Methods

### Criteria for inclusion and exclusion

A study must fulfill the following inclusion criteria to be included in this review:

### Types of studies

Observational cohort studies (with and without control group), cross-sectional studies and randomized clinical trials (RCT). Studies should contain both laboratory and clinical information. Animal studies were excluded.

### Types of participants

Adults, older than 18 years, with sciatica. Inflammatory activity is measured in serum, cerebrospinal fluid (CSF) or in tissues obtained through biopsy.

### Types of outcome measures

For question 1) regarding the presence of biomarkers, the primary outcome was presence of inflammatory proteins in serum, biopsies or CSF. There was no restriction to laboratory methods, including ELISA and Western Blotting for serum and CSF, and messenger RNA qualitative polymerase chain reaction (mRNA qPCR) for biopsy studies.

For question 2) regarding clinical features, the outcomes were pain and physical functional status. The following self-reported outcome measures were assessed: pain intensity (e.g. visual analogue scale (VAS)), back-specific disability (e.g. Roland Morris, Oswestry Disability Index), and perceived recovery (e.g. overall improvement).

### Search methods

A systematic literature review was performed according to the Preferred Reporting Items for Systematic Reviews and Meta-Analysis (PRISMA)-statement [10]. Studies were identified by searching PubMed, Embase.com, Cochrane Central Register of Controlled Trials/Wiley and Web of Science/Clarivate Analytics from inception up to 19 December 2018. The following concepts, including synonyms and closely related words, were used as index terms or free-text words: ‘sciatica’, ‘inflammation’ and ‘cytokines’.

The full search strategy for all databases can be seen in Additional file 1. References of retrieved articles and relevant overview articles were checked to identify additional studies.

## Methods of review

### Study selection

Three authors (MJ/BTM/TVO) independently screened the abstracts and titles retrieved by the search strategy and applied the inclusion criteria. Duplicate articles were excluded. Full texts were obtained if the abstract fulfilled the inclusion criteria and were subsequently screened on inclusion criteria by the authors, independently following the PRISMA guidelines. The checklist can be seen in Additional file 2. Any disagreements between the authors were resolved by discussion and consensus.

### Risk of bias assessment

Two authors (MJ and TVO) independently conducted the risk-of-bias assessment. Risk of Bias (ROB) was evaluated using the Quality in Prognosis Studies (QUIPS) tool [11]. The reason to choose for QUIPS is that in this review we included observational studies assessing the (longitudinal) association between the level of inflammatory activity and clinical symptoms. This resembles very closely a prognostic model and therefore we used the QUIPS tool that supports a systematic appraisal of such studies. It is based on recommendations from a comprehensive review of quality assessment in prognosis systematic reviews and is informed by basic epidemiologic principles. Independently developed and modified versions of the tool have been successfully used by several research groups, with moderate to substantial interrater reliability.

The QUIPS tool considers the following 6 domains of bias: (1) bias due to patient selection (2) attrition, (3) prognostic factor measurement, (4) outcome measurement, (5) study confounding (6) statistical analysis and reporting. Items and operationalization are given in Additional file 3. Due to the explorative nature of this review, only the first four domains were included in the risk of bias assessment. The items of these four domains were each scored to assess the overall risk of bias of the included study. For each item within a domain the responses can be: `yes’, `partial’, `no’ or `unsure’. The responses on these items were combined to assess the risk of bias per domain. The risk of bias for each domain was scored as `high’ (+), `moderate’ (+/−) or `low’ (−) risk of bias. In line with Den Bakker et al. [12], a study was considered to be of low overall risk of bias when the domain scores were rated as low or moderate on all of the 4 domains, with at least 2 rated as low (including the outcome measurement domain). We scored a study as having high overall risk of bias if 2 or more of the domains were judged as high. A study was scored as moderate if the criteria for ‘low’ or ‘high’ were not met. Low overall risk of bias implies that the associations found in this study are unlikely to be different for participants and eligible nonparticipants, not to be different for completing and non completing participants, not to be different for different levels of the outcome of interest, and unlikely to be different related to the baseline level of the prognostic factor [11].

### Data extraction

Data were extracted independently by two review authors (MJ, TVO). The following data were extracted: (1) characteristics of the studies: number of participants, gender, age; (2) characteristics of inflammatory activity (what biomarkers and how they were measured); (3) characteristics of the outcomes: outcome measures, instruments, and scores (e.g. mean, median, standard deviation, and confidence interval). Any disagreements were discussed between the two authors and a third review author (BTM) was consulted if necessary.

### Data analysis and statistics

Due to the heterogeneous data our approach was merely descriptive. For question 1) regarding the presence of biomarkers the type and material (serum/CSF/biopsy) were extracted. For question 2) the measures of association that were presented in the included papers were extracted. For example, the correlation between pain measured by a VAS score and biomarker expression. We present the results of the cross-sectional studies and the longitudinal studies separately. In terms of interpretation we used the following guidance: a correlation coefficient of − 1 or + 1 indicates a perfect linear relation [13]. When Odds Ratio’s (OR) were presented these were extracted, including the *p*-value or the 95% CI and the magnitude of the OR was interpreted as follows: OR = 1.68, 3.47, and 6.71 are equivalent to Cohen’s d = 0.2 (small), 0.5 (medium), and 0.8 (large) [14]. For other measures of association the p-value was used to assess if the association was statistically significant.

## Results

### Description of studies

**Fig. 1:**
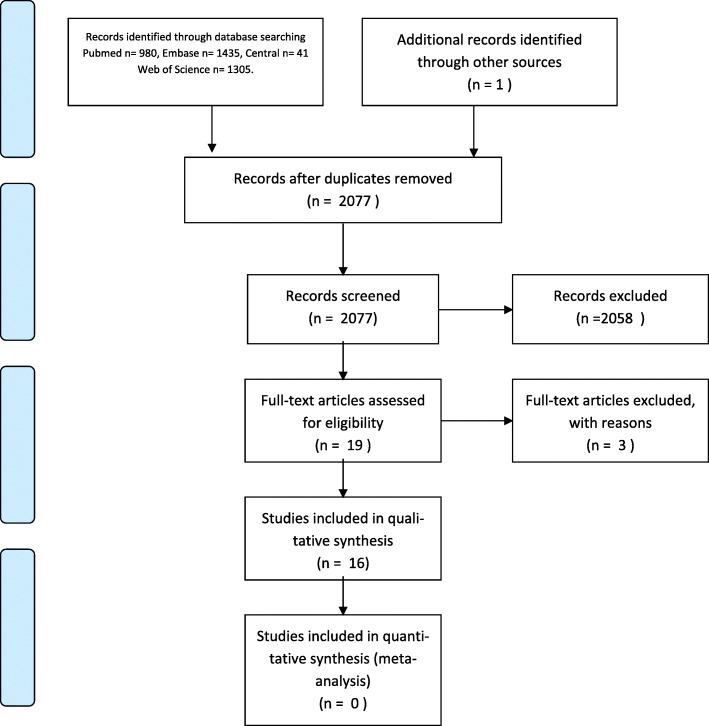
Flowchart of systematic review

The electronic search initially yielded 3761 articles: 980 in PubMed, 1435 in EMBASE, 41 in CENTRAL and 1305 in Web of Science. After de-duplication 2076 articles were left. Of these, 948 were excluded. The main reasons for exclusion were use of animals or conference abstracts. One study by Schistadt et al. [15] was identified through the reference list of Pedersen et al. [26]. Eventually 19 articles fulfilled the criteria for inclusion, of which 16 were analyzed and 3 were excluded. The 16 studies that were analyzed consisted of 7 cross sectional observational studies [16–22] and 9 prospective cohort studies [15, 23–30]. The studies of Kraychete et al., Weber et al. and Miao et al. were excluded because clinical information was lacking [31] or no correlation between biomarkers and clinical outcomes was described [32, 33]. The analyzed studies included a total number of 1212 patients. For overview see flowchart (Fig. 1).

### Risk of Bias (RoB) assessment

The results of the risk of bias assessment are shown in Table 1. Of the cross sectional studies classified as low overall risk of bias [21, 22], and 5 were classified as moderate risk of bias [16–20], mainly due to inadequate participation [16, 17] or moderate outcome reporting [15, 16, 18, 19].

**Table 1:** Results of risk of bias assessment using the adjusted QUIPS-tool

Cross sectional studies					
	Participation	Attrition	Prognostic Factor	Outcome	Risk of bias: + = high +/− = moderate - = low
Piperno 1997 [16]	Moderate	Moderate	Low	Moderate	+/−
Brisby [17]	High	Low	Low	Moderate	+/−
Sugimori [18]	High	Low	Moderate	Low	+/−
Cheng [19]	Moderate	Low	Low	Moderate	+/−
Xue [20]	Moderate	Low	Low	Moderate	+/−
Peng [21]	Moderate	Low	Moderate	Low	–
Palada [22]	Low	Low	Low	Low	–
Longitudinal studies					
Schistadt [15]	Low	Moderate	Low	Low	–
Stürmer [23]	Low	Moderate	Low	Low	–
Andrade [24]	High	Low	Low	Moderate	+/−
Andrade [25]	High	Low	Low	Moderate	+/−
Pedersen [26]	Low	High	Low	Moderate	+/−
Wang [27]	Low	Low	Low	Moderate	–
Moen [28]	Low	Moderate	Low	Low	–
Zu [29]	Low	Moderate	Low	Low	–
Chen [30]	Moderate	Moderate	Low	Moderate	+/−

Of the longitudinal studies, 5 were classified as low high quality [14] risk of bias [15, 23, 27–29] and four were considered as moderate risk of bias [24, 25, 23, 29] mainly due to inadequate participation [25, 26] or high number of drop outs (attrition) [2].

### Biomarkers

The following biomarkers were examined, most of them cytokines (12 of 17 studies): interleukin-1β (IL-1β) [21, 26], interleukin-2 (IL-2) [21], interleukin 4 (IL-4) [21, 30], interleukin-6 (IL-6) [14, 21, 25–27], interleukin-8 (IL-8) [17, 21, 26, 27], interleukin-10 (IL-10) [21, 27], interleukin-17 (IL-17) [19], interleukin-21 (IL-21) [30]. Palada et al. studied a biomarker panel including TNF, interferon-gamma (INFg), IL-1b, IL-2, IL-4, IL-6, IL-8, IL-10, IL-12p70, IL-13 and monocyte chemotactic protein 1 (MCP1) [21]. Three studies measured tumor necrosis factor-α (TNF-α) [25, 28, 20] and one study looked for phospholipase A2 [16]. Sturmer et al. and Sugimori et al. measured levels of high sensitivity C-reactive protein (hsCRP), a sensitive marker of low grade systemic inflammation [18, 23]. Peng et al. looked for expression of the chemokines CX3CL1 and CCL2 [21]. Moen et al. measured 92 different pro and anti-inflammatory cytokines the results of which they compiled in an composite inflammation score [28]: 13 were significantly upregulated, including C-X-C motif chemokine 5 (CXCM5; 217% increase), epidermal growth factor (EGF; 142% increase), and monocyte chemotactic protein 4 (MCP-4; 70% increase).

Thirteen studies measured inflammatory activity in serum [15–23, 26–29], four used biopsies of the nucleus pulposus (NP) [20, 24, 25], annulus fibrosus (AF) [24, 25] and ligamentum flavum (LF) [24]. Two studies used CSF for analysis [17, 22]. The following techniques were used: ELISA [15, 17, 19, 26, 27, 29], mRNA/ qPCR [20, 22, 24], proximal extension assay (PEA) [28], Western Blotting [21, 30]. The two hsCRP studies used latex agglutination [18, 23].

### Clinical features in relationship to biomarkers

Tables 2 and 3 summarize the duration of symptoms, age), type of marker and sampling, the clinical parameters and associations between biomarkers and clinical parameters that were found. We distinguished between cross sectional studies (Table 2) and longitudinal studies (Table 3) studies.

**Table 2:** Inflammatory biomarkers in relationship to clinical features (cross sectional studies)

Study	Age (yr)	Duration (months)	Source	Technique	Marker	Clin O	Ass
Piperno [16]	40 + − 13	20 + −26	serum	Degradation	PhosA2	VAS	no
Brisby [17]	N	92 (5–390)^a^	serum CSF	ELISA	Il-8	VAS	r = −0,48
Sugimor [18]	26.4 (16–39)	N	serum	Latex agl	hsCRP	JOA	r = −0,583
Cheng [19]	44 (30–72)	N	serum	ELISA	Il-17	VAS	r = 0,458
Xue [20]	52 (21–70)	N	serum NP Biopsy	mRNA qPCR	Il-21	VAS	r = 0,809
Peng [21]	34.2 (+ − 5.8)^b^	4.5 (1–22)	serum	Western blot	CX3CL1	VAS	r = 0, 393
			serum	Western blot	CX3CL1	JOA	r = − 0,342
			serum	Western blot	CCL2	VAS	r = 0, 360
			serum	Western blot	CCL2	JOA	r = −0,375
Palada [22]	41.13 (15–65)	> 1 month	serum	mRNA qPCR	Il-6	VAS	r = 0,380
			CSF	mRNA qPCR	Il-8	VAS	r = 0,395
			serum	mRNA qPCR	MCP1	VAS	r = 0,515

*Ass* association, *Clin O* clinical outcome, *CSF* cerebrospinal fluid, *ELISA* enzyme linked serum assay, *IL* interleukin, *JOA* Japanese orthopedic association score, *Latex agl* latex agglutination, *NP* nucleus pulposus, *qPCR* quantitative polymerase chain reaction, *VAS* visual analogue scale, *Yr* years

^a^days

^b^VAS > 7

**Table 3:** Inflammatory biomarkers in relationship to clinical features (longitudinal studies)

Study	Age (yr)	Duration (weeks)	Source	Technique	Substance	Clin O	Ass
Schistad [15]	41.3 (10)	20.3 (19.9)	serum	ELISA	Il-6	VAS	B = 0,64^a^
Sturmer [23]	44.8 (12.4)	acute^b^	serum	latex agl	hsCRP	VAS > 4	aOR = 3,4^c^
Andrade [24]	49	N	NP Biopsy	mRNA qPCR	TNFa	VAS	r = 0,65
			AF Biopsy	mRNA qPCR	TNFa	VAS	r = 0,06
			LF Biopsy	mRNA qPCR	TNFa	VAS	r = 0,29
Andrade [25]	41	13–26	NP Biopsy	mRNA qPCR	IL-6	VAS	r = 0,23
			NP Biopsy	mRNA qPCR	IL-1b	VAS	r = 0,05
			NP Biopsy	mRNA qPCR	IL-6	VAS	r = −0,11
			NP Biopsy	mRNA qPCR	IL-1b	VAS	r = 0,03
Pedersen [26]	39.3 (18–58)	32.3 + − 4.5	serum	ELISA	IL-6	VAS	F(1.0, 118) = 9,7
			serum	ELISA	IL-8	VAS	F(1.0, 118,0) = 6,9
Wang [27]	37 + −13.3)^d^	48 (+ − 29)	serum	ELISA	IL-6	ODI	r = 0,394
			serum	ELISA	TNFa	ODI	r = 0,629
			serum	ELISA	IL-10	ODI	r = 0,415
			serum	ELISA	IL-8	ODI	r = −0,133
Moen [28]	40 (9)	> 8	serum	PEA	inflammation score	VAS	positive Linear discriminant analysis
Zu [29]	34.0 + −12.3^e^	48 (+ − 29)^c^	serum	ELISA	TNFa	ODI + VAS > 3	r = 0,2
			serum	ELISA	TNFa	ODI + VAS < 3	r = 0,37
			serum	ELISA	IL-4	ODI + VAS > 3	r = 0,09
			serum	ELISA	IL-4	ODI + VAS < 3	r = 0,08
Chen [30]	51.3 + −24.4	N	NP biopsy	Western blot	IL-21	VAS	r = 0.834

*AF* annulus fibrosus, *aOR* adjusted Odds Ratio, *Ass* association, *Clin O* clinical outcome, *LF* ligamentum flavum, *ELISA* enzyme linked immune assay, *hsCRP* high sensitive c-reactive protein, *Il* interleukin, *latex agl* latex agglutination, *mRNA* messenger RNA, *N* unknown, *NP* nucleus pulposus, *ODI* Oswesty Disability Index, *PEA* proximal extension assay, *TNF* tumour necrosis factor alpha, *Yr* years, *VAS* visual analogue scale

^a^multivariate regression analysis

^b^no definition

^c^adjusted for age, sex, smoking, alcohol, body mass, use of diuretics and analgetic drugs and steroid injections during the previous 24 h

^d^high pain group (VAS > 3)

^e^subgroup ruptured AF

All studies included patients who suffered from sciatica for more than 3 months (average), and therefor had chronic low back pain. All studies reported VAS (Visual analog scale) as assessment tool for pain, except Sugimori et al. and Wang et al. [18, 27]. Piperno et al. also used the Dallas Pain Questionnaire [16]. Pain duration at baseline was described precisely in 2 of the cross sectional studies [17, 21] and 4 of the longitudinal studies [15, 26, 27, 29]. Wang et al., determined functioning using the Oswestry Disability Index (ODI) and also used the short form-36 (SF-36) questionnaire [27]. Sugimori et al. and Peng et al. also used the Japanese Orthopedic Association Score for overall functioning.^187,21^Most of the associations between markers and clinical symptoms, were found in the serum studies using ELISA techniques.

For the cross sectional studies a strong positive correlation was found between IL-21 and VAS for pain in one study (r = 0,809 [20]. A moderate positive correlation was found for MCP-1 in serum (r = 0,659) [22] and hsCRP in serum (r = 0,538) [18]. The moderate negative correlation between the JOA score and hsCRP. should be explained positively as a high JOA score implies better clinical functioning.

For the longitudinal studies a strong positive correlation was found between Il-21 and VAS for pain in one study (r = 0,834) [30]. A moderate positive correlation was found for TNF-a in both serum (r = 0,629) [27] and biopsy (r = 0.65) [24]. For IL-8 in [2] and Il-6 in annulus fibrosis biopsy [27] low negative correlations were found: the presence of these markers is related to better clinical outcome. Moen et al. calculated an inflammation score (a weighted average of 41 protein scores) that was positive for all high pain patients (VAS > 40)^287^. Sturmer et al. showed that severe pain (VAS > 4) is associated with increased hsCRP levels among patients with sciatica (adjusted OR = 3.4 (95% CI, 1.1 to 10) [23]. Corrections were made for age, sex, smoking and alcohol consumption. The prospective data of Pedersen et al. showed that levels IL-6 and IL-8 in serum were related to pain intensity measured on a VAS (IL-6, F(1.0, 118) = 9.7, *p* = 0.002 test of between subjects effect; IL-8, F(1.0, 118.0) = 6.9, *p* = 0.01 test of between subjects effect, rmANOVA, covariates age for IL-6; smoking for Il-6 and Il-8; and treatment for IL-8 [26]. In their multivariate analysis Schistadt el al showed that high levels of serum IL-6 correlated with high VAS for leg pain (beta score 0,64) and accounted for 25% of the variance in the VAS for leg pain at 1-year follow-up [15]. Schistadt et al. concluded that in addition to elevated Il-6 levels, intense pain, long surgery wait and low education are related to slow recovery [15]. The other studies did not give detailed information about the patients and their history in terms of education, work status, previous back surgery, comorbidity or the medication that was used.

## Discussion

The studies under review were heterogeneous with regard to the population, the biomarkers that were studied and the laboratory methods that were used. For that reason pooling of data (meta-analysis) was impossible. The overall Risk of Bias (as assessed by the adapted QUIPS-tool) was moderate 9/12 studies; participation and measurement of the clinical outcome in particular were not optimal. Most frequently the VAS was used for the measurement of pain, but the studies did not accurately describe the location of the pain (back or leg) the reference point (i.e. time-window) or type of pain (for example average pain on activity or during the day). Therefore it is hard to draw firm conclusions, and although the strong positive correlation between IL-21 and pain in two studies [20, 30], and the association between hsCRP levels and severe pain (VAS > 40) [23] might be of interest, they should be interpreted with great care.

### Strengths and limitations

A strength of this study is the systematic and transparent approach that was followed in all the steps of this systematic review.

Still several biases can be introduced by literature search and selection procedure. First, due to selection bias relevant publications may have been missed. For example in our initial search we missed the relevant publication by Schistadt et al. [15]. Second, due to publication bias unpublished studies may have been missed. Third there might be reference bias: screening references may result in an over representation of positive studies, as trials with a negative result are less likely to be referred to.

Another limitation is that we used an adjusted version of the QUIPS-tool to asses ROB. We did not take into account the domains ‘study confounding’ and ‘statistical analysis out’. We did not find relevant information in the literature to decide a-priori which confounders would be the most relevant in this field. Still, where possible, in the result section where we describe which factors were taken into account in the included studies. But unfortunately many studies no detailed information was included about other factors they took into account.

### Implications for practice

The results of this review are not overly convincing which may suggest only a minor role for inflammation in sciatica. Of course this is based on limited data, however these results could potentially be interpreted in line with the results from therapeutic studies. There are two interventions in patients with sciatica, targeted at inflammation: 1) use of non steroidal anti inflammatory drugs (NSAIDs); 2) epidural injections with corticosteroids. The effects of both NSAIDs and injections seem to be minor.

A Cochrane review showed very low-quality evidence that the efficacy of NSAIDs for pain reduction is comparable with that of placebo and low-quality evidence that NSAIDs is better than placebo for global improvement [34].

With regard to effectivity of epidural corticosteroid injections a meta-analysis of 23 trials [35] showed a small positive short-term (< 3 months) effect for leg pain of epidural corticosteroid injections compared to placebo (mean difference (MD), − 6.2 on a 100 points VAS) [95% CI, − 9.4 to − 3.0]) and disability (MD, − 3.1 on a 100 point Oswestry Disability Scale). A second meta-analysis of 30 trials [36] showed an immediate-term (< 2 weeks) pain reduction (MD − 7.55 on a 100 point VAS [95% CI, − 11.4 to − 3.74]) and reduction in disability (standardized MD, − 0.33 [95% CI, − 0.56 to − 0.09]) of epidural corticosteroid injections compared to placebo.

A potential explanation for a lack of treatment effect of both NSAIDs and epidural corticosteroid injections could be that inflammation plays a minor role in sciatica, or only plays an important role in a small subgroup of patients. Perhaps in the future we can identify patients with sciatica that respond well to both treatments for example acute patients (that were underrepresented in this systematic review) or patients with severe pain.

To summarize: though anti-inflammatory treatment (in the form of NSAIDs or epidural injections with corticosteroids) is the first choice of pain treatment in patients with sciatica, the evidence of inflammation playing a role in sciatica is not overly convincing based on laboratory studies.

### Implications for research

The main question to be still answered here is if inflammation plays a role in lumbar radicular syndrome, at what stage and to what extent? From a research perspective, we think that the acute stage of sciatica (< 12 weeks) deserves more attention given that the fact that although most patients recover within this period [37]. During the acute stage serum studies are relatively easy to perform. It is interesting to know what specific cytokines are elevated and if they have a prognostic value e.g. for chronicity. The markers that had high correlations with clinical measures in previous studies (for example Il-21) seem the most interesting candidates for further study. In addition we think that different laboratories should come to a consensus regarding the best method for measuring inflammation in sciatica.

In the nearby future inflammatory biomarkers could possibly predict the clinical course of sciatica and be used to identify subsets of patients that respond best to anti-inflammatory treatment (NSAIDs or epidural injections with corticosteroids) or patients that benefit from surgery.

## Conclusion

In this systematic review there was considerable heterogeneity in the type of biomarkers and in the clinical measurements in the included studies. Taking into account the overall risk of bias of the included studies there is insufficient evidence to draw firm conclusions regarding the relationship between inflammation and clinical symptoms in patients with sciatica.

## Additional files

Additional file 1: The full search strategy for all databases. (DOCX 30 kb)

Additional file 2: Prisma Checklist for reporting in systematic reviews and meta-analyses. (DOC 62 kb)

Additional file 3: The Quality in Prognosis Studies Tool (QUIPS). (DOCX 17 kb)

## Abbreviations

AF: Annulus fibrosus
aOR: Adjusted Odds ratio
CI: Confidence interval
CSF: Cerebrospinal fluid
CXCM5: C-X-C motif chemokine 5
EGF: Epidermal growth factor
Elisa: Enzyme-linked immunosorbent assay
hsCRP: High sensitive C-reactive protein
IL: Interleukin
INFg: Interferon gamma
JOA: Japanese orthopedic association (score)
LF: Ligamentum flavum
MCP-4: Monocyte chemotactic protein-4
mRNA qPCR: Messenger RNA qualitative polymerase chain reaction
NP: Nucleus pulposus
NSAIDS: Non-steroidal anti-inflammatory drugs
ODI: Oswestry Disability Index
PEA: Proximal extension assay
Quips: Quality in prognosis studies
RCT: Randomized controlled trial
SF-36: Short form 36
VAS: Visual analogue scale
