# Supplementary material for: Inflammatory biomarkers in patients with sciatica: a systematic review
Source: BMC Musculoskelet Disord. 2019 Apr 9;20:156. doi: 10.1186/s12891-019-2541-0 (PMC6456959; doi:10.1186/s12891-019-2541-0)
Supplement: Supplementary file 1 — The full search strategy for all databases. (DOCX 30 kb) [file 12891_2019_2541_MOESM1_ESM.docx]

PubMed.com, 19-12-2018

| **No.** | **Query** | **Results** |
| --- | --- | --- |
| #4 | Search (#1 AND #2 AND #3) | 980 |
| #3 | Search "Inflammation"[Mesh] OR inflamm*[tiab] OR proinflamm*[tiab] OR antiinflamm*[tiab] | 1027443 |
| #2 | Search "Cytokines"[Mesh] OR Cytokine*[tiab] OR Interferon*[tiab] OR IFN-alpha*[tiab] OR Interleukin*[tiab] OR IL-1[tiab] OR IL1[tiab] OR T Helper Factor*[tiab] OR Lymphocyte Activating Factor*[tiab] OR Macrophage Cell Factor*[tiab] OR Epidermal Cell Derived Thymocyte-Activating Factor*[tiab] OR Hematopoietin-1[tiab] OR Catabolin[tiab] OR IL-2[tiab] OR IL2[tiab] OR TCGF[tiab] OR Lymphocyte Mitogenic Factor*[tiab] OR T-Cell Growth Factor*[tiab] OR Thymocyte Stimulating Factor*[tiab] OR Ro-23-6019[tiab] OR Ro236019[tiab] OR Ro-236019[tiab] OR RU 49637[tiab] OR RU49637[tiab] OR IL-3[tiab] OR IL3[tiab] OR Mast-Cell Colony-Stimulating Factor*[tiab] OR Multipotential Colony-Stimulating Factor*[tiab] OR P-Cell Stimulating Factor*[tiab] OR Erythrocyte Burst-Promoting Factor*[tiab] OR Hematopoietin-2[tiab] OR IL-4[tiab] OR IL4[tiab] OR B-Cell Stimulating Factor*[tiab] OR BCGF-1[tiab] OR Binetrakin[tiab] OR BSF-1[tiab] OR Mast Cell Growth Factor-2[tiab] OR MCGF-2[tiab] OR B Cell Stimulatory Factor-1[tiab] OR B-Cell Growth Factor*[tiab] OR B-Cell Proliferating Factor*[tiab] OR B-Cell Stimulatory Factor 1[tiab] OR IL-5[tiab] OR IL5[tiab] OR BCGF-II[tiab] OR Eosinophil Differentiation Factor*[tiab] OR T-Cell Replacing Factor*[tiab] OR T-Cell-Replacing Factor*[tiab] OR B-Cell Growth Factor-II[tiab] OR IL-6[tiab] OR IL6[tiab] OR Differentiation Factor 2, B Cell[tiab] OR IFN-beta 2[tiab] OR MGI-2[tiab] OR Myeloid Differentiation-Inducing Protein*[tiab] OR Plasmacytoma Growth Factor*[tiab] OR B Cell Stimulatory Factor-2[tiab] OR B-Cell Differentiation Factor[tiab] OR B-Cell Stimulatory Factor-2[tiab] OR BSF-2[tiab] OR Hepatocyte-Stimulating Factor*[tiab] OR Hybridoma Growth Factor*[tiab] OR IL-7[tiab] OR IL7[tiab] OR Lymphopoietin*[tiab] OR IL-8[tiab] OR IL8[tiab] OR Monocyte-Derived Neutrophil-Activating Peptide*[tiab] OR Anionic Neutrophil-Activating Peptide[tiab] OR Chemokine CXCL8[tiab] OR CXCL8 Chemokine*[tiab] OR Macrophage-Derived Chemotactic Factor*[tiab] OR Neutrophil Chemotactic Factor*[tiab] OR Neutrophil Activation Factor*[tiab] OR Granulocyte Chemotactic Peptide-Interleukin-8[tiab] OR Monocyte-Derived Neutrophil Chemotactic Factor*[tiab] OR Lymphocyte-Derived Neutrophil-Activating Peptide*[tiab] OR Alveolar Macrophage Chemotactic Factor-I[tiab] OR AMCF-I[tiab] OR IL-9[tiab] OR IL9[tiab] OR T-Cell Growth Factor P40[tiab] OR P40 T-Cell Growth Factor*[tiab] OR IL-10[tiab] OR IL10[tiab] OR CSIF-10[tiab] OR Cytokine Synthesis Inhibitory Factor*[tiab] OR IL-11[tiab] OR IL11[tiab] OR Adipogenesis Inhibitory Factor*[tiab] OR IL-12[tiab] OR IL12[tiab] OR Edodekin Alfa[tiab] OR Natural Killer Cell Stimulatory Factor*[tiab] OR Cytotoxic Lymphocyte Maturation Factor[tiab] OR IL-12p35[tiab] OR IL12p35[tiab] OR IL-12p40[tiab] OR IL12p40[tiab] OR IL-13[tiab] OR IL13[tiab] OR IL-15[tiab] OR IL15[tiab] OR IL-16[tiab] OR IL16[tiab] OR Lymphocyte Chemoattractant Factor*[tiab] OR LCF Factor*[tiab] OR IL-17*[tiab] OR IL17*[tiab] OR CTLA-8[tiab] OR CTLA8[tiab] OR Cytotoxic T lymphocyte-Associated Antigen 8[tiab] OR IL-18[tiab] OR IL18[tiab] OR IFN-gamma-Inducing Factor*[tiab] OR IL-23[tiab] OR IL23[tiab] OR IL-23p19[tiab] OR IL-23 p19[tiab] OR IL23 p19[tiab] OR IL23p19[tiab] OR IL-27[tiab] OR IL27[tiab] OR Monokine*[tiab] OR Tumor Necrosis Factor*[tiab] OR TNF[tiab] OR TNF Receptor Ligand*[tiab] OR TNFalpha[tiab] OR TNF-alpha[tiab] OR Cachectin[tiab] OR TNF Superfamily[tiab] OR TNF[tiab] OR "Biomarkers"[Mesh] OR biomark*[tiab] OR serum mark*[tiab] OR biological mark*[tiab] | 1583200 |
| #1 | Search "Intervertebral Disc Displacement"[Mesh] OR "Sciatica"[Mesh] OR Sciatic*[tiab] OR slipped dis*[tiab] OR Intervertebral Disc Displacement*[tiab] OR Intervertebral Disk Displacement*[tiab] OR herniated dis*[tiab] OR prolapsed dis*[tiab] OR radicular pain*[tiab] OR disc herniation*[tiab] OR disk herniation*[tiab] | 49574 |

Embase.com, 19-12-2018

| **No.** | **Query** | **Results** |
| --- | --- | --- |
| #11 | #3 AND #6 AND #10 | **1435** |
| #10 | #7 OR #8 OR #9 | **1903023** |
| #9 | 'biological marker'/exp OR biomark*:ab,ti OR 'serum mark*':ab,ti OR 'biological mark*':ab,ti OR bioindicat*:ab,ti | **420964** |
| #8 | 'cytokine*':ab,ti OR 'interleukin*':ab,ti OR 'il1*':ab,ti OR 'il 1':ab,ti OR ((leucocyt* NEXT/1 pyrogen):ab,ti) OR ((leukocyt* NEXT/1 pyrogen):ab,ti) OR 'leukocytic endogenous mediator':ab,ti OR 'lymphocyte activating factor':ab,ti OR 'hemopoietin 1':ab,ti OR 'beta interferon inducing 22 k factor':ab,ti OR 'beta inter-feron inducing 22k factor':ab,ti OR 'interferon beta inducing 22k factor':ab,ti OR 'il2*':ab,ti OR 'il 2':ab,ti OR 'bioleukin':ab,ti OR 'lymphocult t hp':ab,ti OR 'lymphocyte mitogenic factor':ab,ti OR 't cell growth factor':ab,ti OR 't cell growth factor 2':ab,ti OR 't lymphocyte growth factor':ab,ti OR 't lymphocyte growth factor 2':ab,ti OR 'il3*':ab,ti OR 'il 3':ab,ti OR (('haematopoietic cell growth' NEXT/1 factor*):ab,ti) OR (('hematopoietic cell growth' NEXT/1 factor*):ab,ti) OR (('haemopoietic cell growth' NEXT/1 factor*):ab,ti) OR (('hemopoietic cell growth' NEXT/1 factor*):ab,ti) OR 'hemopoietin 2':ab,ti OR 'mast cell growth factor':ab,ti OR 'mast cell growth factor 2':ab,ti OR ((multi* NEXT/1 'colony stimulating factor'):ab,ti) OR 'p cell stimulating factor':ab,ti OR 'il4*':ab,ti OR 'il 4':ab,ti OR 'b cell stimulating factor*':ab,ti OR 'b cell stimulatory factor*':ab,ti OR 'b lympho-cyte stimulating factor 1':ab,ti OR 'bsf 1':ab,ti OR 'bsf1':ab,ti OR 'eosinophil differentiation factor':ab,ti OR 'il5*':ab,ti OR 'il 5':ab,ti OR 'b cell growth factor*':ab,ti OR 'killer helper factor':ab,ti OR 't cell replacing fac-tor':ab,ti OR 't lymphocyte replacing factor':ab,ti OR 'il6*':ab,ti OR 'il 6':ab,ti OR '26 k protein':ab,ti OR (('b cell' NEXT/1 stimulat* NEXT/1 'factor 2'):ab,ti) OR 'b lymphocyte stimulating factor 2':ab,ti OR (('beta 2' NEAR/1 interferon):ab,ti) OR (('beta2' NEAR/1 interferon):ab,ti) OR 'bsf 2':ab,ti OR 'bsf2':ab,ti OR 'hepatocyte stimulating factor':ab,ti OR 'liver cell stimulating factor':ab,ti OR 'plasmacytoma growth factor':ab,ti OR 'protein 26k':ab,ti OR 'il7*':ab,ti OR 'il 7':ab,ti OR 'lymphopoietin 1':ab,ti OR 'pre b cell growth factor':ab,ti OR 'pre b lymphocyte growth factor':ab,ti OR 'il8*':ab,ti OR 'il 8':ab,ti OR ((chemokine NEAR/1 cxcl8):ab,ti) OR 'cxc chemokine ligand 8':ab,ti OR 'granulocyte chemotactic peptide':ab,ti OR 'lymphocyte derived neutrophil activating peptide':ab,ti OR 'lynap':ab,ti OR 'monap':ab,ti OR 'monocyte derived neutrophil activating peptide':ab,ti OR 'monocyte derived neutrophil chemotactic factor':ab,ti OR 'neutrophil activating factor':ab,ti OR 'neutrophil activating peptide':ab,ti OR 'neutrophil attracting peptide':ab,ti OR 'polymorphonuclear granulocyte activating factor':ab,ti OR 'il9*':ab,ti OR 'il 9':ab,ti OR 'il10*':ab,ti OR 'il 10':ab,ti OR 'cytokine synthesis inhibitory factor':ab,ti OR 'csif':ab,ti OR 'il12*':ab,ti OR 'il 12':ab,ti OR 'cytotoxic lymphocyte maturation factor':ab,ti OR 'clmf':ab,ti OR 'natural killer cell stimulatory factor':ab,ti OR 'nksf':ab,ti OR 'cytotoxic lymphocyte maturation factor 2':ab,ti OR 'natural killer cell stimulatory factor 2':ab,ti OR 'il13*':ab,ti OR 'il 13':ab,ti OR 'il15*':ab,ti OR 'il 15':ab,ti OR 'il16*':ab,ti OR 'il 16':ab,ti OR 'lymphocyte chemoattractant factor':ab,ti OR 'il17*':ab,ti OR 'il 17':ab,ti OR 'cytotoxic t lymphocyte antigen 8':ab,ti OR 'cytotoxic t lymphocyte associated antigen 8':ab,ti OR 'cytotoxic t lymphocyte associated protein 8':ab,ti OR 'cytotoxic t lymphocyte protein 8':ab,ti OR 'ctla 8':ab,ti OR 'ctla8':ab,ti OR 'il18*':ab,ti OR 'il 18':ab,ti OR 'gamma interferon inducing factor':ab,ti OR 'igif':ab,ti OR 'interferon gamma inducing factor':ab,ti OR 'cutaneous t cell attracting chemokine':ab,ti OR 'cc chemokine ligand 27':ab,ti OR 'ccl27':ab,ti OR 'ctack':ab,ti OR 'scya27':ab,ti OR 'small inducible cytokine a27':ab,ti OR 'eotaxin*':ab,ti OR 'ccl11':ab,ti OR 'fibroblast growth factor*':ab,ti OR 'fibroblast stimulating factor':ab,ti OR 'heparin binding growth factor':ab,ti OR 'granulocyte colony stimulating factor':ab,ti OR 'granulocyte colony-stimulating factor':ab,ti OR 'g csf':ab,ti OR 'granulocyte macrophage colony stimulating factor':ab,ti OR 'granulocyte-macrophage colony-stimulating factor':ab,ti OR 'gm csf':ab,ti OR 'gmcsf':ab,ti OR 'alpha2 interferon':ab,ti OR 'alpha 2 interferon':ab,ti OR 'berofor alpha2':ab,ti OR 'berofor alpha 2':ab,ti OR 'ifn alpha2':ab,ti OR 'ifn alpha 2':ab,ti OR 'interferon alpha ii':ab,ti OR 'interferon alpha2':ab,ti OR 'interferon alpha 2':ab,ti OR 'gamma interferon':ab,ti OR 'human immune interferon':ab,ti OR 'ifn gamma':ab,ti OR 'imunomax gamma':ab,ti OR 'interferon 2':ab,ti OR 'interferon gamma':ab,ti OR 'interferon ii':ab,ti OR 'interferon type ii':ab,ti OR 'interferon-gamma':ab,ti OR 'oh 6000':ab,ti OR 'leukemia inhibitory factor':ab,ti OR (('leukaemia inhibit*' NEXT/1 factor):ab,ti) OR 'cholinergic differentiation factor':ab,ti OR 'macrophage inflammatory protein 1':ab,ti OR 'macrophage inflammatory protein-1':ab,ti OR 'bb 10010':ab,ti OR 'bb10010':ab,ti OR 'cc chemokine ligand 3':ab,ti OR ((ccl3 NEAR/1 chemokine):ab,ti) OR 'ld78':ab,ti OR 'lym-phokine mip 1alpha':ab,ti OR 'mip 1alpha':ab,ti OR ((protein NEAR/1 scya3):ab,ti) OR 'small inducible cytokine a3':ab,ti OR 'cc chemokine ligand 4':ab,ti OR ((ccl4 NEAR/1 chemokine):ab,ti) OR ((scya4 NEAR/1 protein):ab,ti) OR 'small inducible cytokine a4':ab,ti OR 'mip 1beta':ab,ti OR 'platelet derived growth factor':ab,ti OR 'pdgf bb':ab,ti OR 'rantes':ab,ti OR 'cc chemokine ligand 5':ab,ti OR ((chemokine NEAR/1 ccl5):ab,ti) OR 'stem cell factor':ab,ti OR 'c kit ligand':ab,ti OR 'kit ligand':ab,ti OR 'steel factor':ab,ti OR 'stromal cell derived factor 1alpha':ab,ti OR 'sdf 1alpha':ab,ti OR 'stromal cell-derived factor-1alpha':ab,ti OR 'stromal derived factor 1alpha':ab,ti OR 'tumor necrosis factor alpha':ab,ti OR 'mhr 24':ab,ti OR 'tnf alfa':ab,ti OR 'tnf alpha':ab,ti OR 'tumor necrosis factor alfa':ab,ti OR 'tumor necrosis factor-alpha':ab,ti OR 'tumour necrosis factor alfa':ab,ti OR 'tumour necrosis factor alpha':ab,ti OR 'tumour necrosis factor-alpha':ab,ti OR 'alpha lymphotoxin':ab,ti OR 'human tumor necrosis factor beta':ab,ti OR 'human tumour necrosis factor beta':ab,ti OR 'lymphotoxic factor':ab,ti OR 'lymphotoxin alpha':ab,ti OR 'lymphotoxin-alpha':ab,ti OR 'tumor necrosis factor beta':ab,ti OR 'tumour necrosis factor beta':ab,ti OR 'tumor necrosis factor related apoptosis inducing ligand':ab,ti OR ((antigen NEAR/1 cd253):ab,ti) OR (((protein NEAR/1 tnfsf):ab,ti) AND 10:ab,ti) OR ((protein NEAR/1 tnfsf10):ab,ti) OR ((protein NEAR/1 trail):ab,ti) OR 'tnf related apoptosis inducing ligand':ab,ti OR 'tnf-related apoptosis-inducing ligand':ab,ti OR 'tumor necrosis factor ligand superfamily member 10':ab,ti OR 'tumor necrosis factor superfamily member 10':ab,ti OR 'tumour necrosis factor ligand superfamily member 10':ab,ti OR 'tumour necrosis factor related apoptosis inducing ligand':ab,ti OR 'tumour necrosis factor superfamily member 10':ab,ti | **830649** |
| #7 | 'cytokine'/exp | **1412739** |
| #6 | #4 OR #5 | **3718370** |
| #5 | inflamm*:ab,ti OR proinflamm*:ab,ti OR antiinflamm*:ab,ti | **1210995** |
| #4 | 'inflammation'/exp | **3249455** |
| #3 | #1 OR #2 | **62973** |
| #2 | sciatic*:ab,ti OR ischias:ab,ti OR ischiatic:ab,ti OR 'slipped dis*':ab,ti OR 'intervertebral disc displacement*':ab,ti OR 'intervertebral disk displacement*':ab,ti OR 'prolapsed dis*':ab,ti OR 'radicular pain*':ab,ti OR ((dis* NEAR/2 hernia*):ab,ti) OR ((dis* NEXT/1 prolapse*):ab,ti) OR ((dis* NEXT/1 protrusion*):ab,ti) OR 'hernia disci':ab,ti OR 'hernia nuclei pulposi':ab,ti OR 'herniated intervertebral dis*':ab,ti OR 'herniated nucleus pulpos*':ab,ti OR ((dis* NEXT/1 rupture*):ab,ti) OR 'nucleus pulposus hernia*':ab,ti | **52275** |
| #1 | 'intervertebral disk hernia'/exp OR 'sciatica'/exp | **25429** |

CENTRAL, 19-12-2018

| **No.** | **QuerySearch** | **Results** |
| --- | --- | --- |
| #1 | MeSH descriptor: [Intervertebral Disc Displacement] explode all trees | 786 |
| #2 | MeSH descriptor: [Sciatica] explode all trees | 275 |
| #3 | (Sciatic* or slipped dis* or Intervertebral Disc Displacement* or Intervertebral Disk Displacement* or herniated dis* or prolapsed dis* or radicular pain* or disc herniation* or disk herniation*):ti,ab,kw | 3300 |
| #4 | {OR #1-#3} | 3300 |
| #5 | MeSH descriptor: [Inflammation] explode all trees | 9267 |
| #6 | (inflamm* or proinflamm* or antiinflamm*):ti,ab,kw | 62747 |
| #7 | {OR #5-#6} | 66752 |
| #8 | MeSH descriptor: [Biomarkers] explode all trees | 18524 |
| #9 | MeSH descriptor: [Cytokines] explode all trees | 18545 |
| #10 | (biomark* or serum NEXT mark* or biological NEXT mark*):ti,ab,kw | 30481 |
| #11 | (Cytokine* OR Interferon* OR IFN NEXT alpha* OR Interleukin* OR IL-1 OR IL1 OR "T Helper" NEXT Factor* OR "Lymphocyte Activating" NEXT Factor* OR "Macrophage Cell" NEXT Factor* OR "Epidermal Cell Derived Thymocyte-Activating" NEXT Factor* OR Hematopoietin-1 OR Catabolin OR IL-2 OR IL2 OR TCGF OR "Lymphocyte Mitogenic" NEXT Factor* OR "T-Cell Growth" NEXT Factor* OR "Thymocyte Stimulating" NEXT Factor* OR "Ro-23-6019" OR Ro236019 OR "Ro-236019" OR "RU 49637" OR RU49637 OR "IL-3" OR IL3 OR "Mast-Cell Colony-Stimulating" NEXT Factor* OR "Multipotential Colony-Stimulating" NEXT Factor* OR "P-Cell Stimulating" NEXT Factor* OR "Erythrocyte Burst-Promoting" NEXT Factor* OR Hematopoietin-2 OR "IL-4" OR IL4 OR "B-Cell Stimulating" NEXT Factor* OR "BCGF-1" OR Binetrakin OR "BSF-1" OR "Mast Cell Growth Factor-2" OR "MCGF-2" OR B Cell Stimulatory Factor-1 OR "B-Cell Growth" NEXT Factor* OR "B-Cell Proliferating" NEXT Factor* OR B-Cell Stimulatory Factor 1 OR IL-5 OR IL5 OR BCGF-II OR "Eosinophil Differentiation" NEXT Factor* OR "T-Cell Replacing" NEXT Factor* OR "T-Cell-Replacing" NEXT Factor* OR B-Cell Growth Factor-II OR IL-6 OR IL6 OR Differentiation Factor 2, B Cell OR IFN-beta 2 OR MGI-2 OR "Myeloid Differentiation-Inducing" NEXT Protein* OR "Plasmacytoma Growth" NEXT Factor* OR B Cell Stimulatory Factor-2 OR B-Cell Differentiation Factor OR B-Cell Stimulatory Factor-2 OR BSF-2 OR "Hepatocyte-Stimulating" NEXT Factor* OR "Hybridoma Growth" NEXT Factor* OR IL-7 OR IL7 OR Lymphopoietin* OR IL-8 OR IL8 OR "Monocyte-Derived Neutrophil-Activating" NEXT Peptide* OR Anionic Neutrophil-Activating Peptide OR Chemokine CXCL8 OR "CXCL8" NEXT Chemokine* OR "Macrophage-Derived Chemotactic" NEXT Factor* OR "Neutrophil Chemotactic" NEXT Factor* OR "Neutrophil Activation" NEXT Factor* OR Granulocyte Chemotactic Peptide-Interleukin-8 OR "Monocyte-Derived Neutrophil Chemotactic" NEXT Factor* OR "Lymphocyte-Derived Neutrophil-Activating" NEXT Peptide* OR Alveolar Macrophage Chemotactic Factor-I OR AMCF-I OR IL-9 OR IL9 OR T-Cell Growth Factor P40 OR "P40 T-Cell Growth" NEXT Factor* OR IL-10 OR IL10 OR CSIF-10 OR "Cytokine Synthesis Inhibitory" NEXT Factor* OR IL-11 OR IL11 OR "Adipogenesis Inhibitory" NEXT Factor* OR IL-12 OR IL12 OR Edodekin Alfa OR "Natural Killer Cell Stimulatory" NEXT Factor* OR Cytotoxic Lymphocyte Maturation Factor OR IL-12p35 OR IL12p35 OR IL-12p40 OR IL12p40 OR IL-13 OR IL13 OR IL-15 OR IL15 OR IL-16 OR IL16 OR "Lymphocyte Chemoattractant" NEXT Factor* OR "LCF" NEXT Factor* OR IL-17* OR IL17* OR CTLA-8 OR CTLA8 OR Cytotoxic T lymphocyte-Associated Antigen 8 OR IL-18 OR IL18 OR "IFN-gamma-Inducing" NEXT Factor* OR IL-23 OR IL23 OR IL-23p19 OR IL-23 p19 OR IL23 p19 OR IL23p19 OR IL-27 OR IL27 OR Monokine* OR "Tumor Necrosis" NEXT Factor* OR TNF OR "TNF Receptor" NEXT Ligand* OR TNFalpha OR TNF-alpha OR Cachectin OR TNF Superfamily OR TNF):ti,ab,kw | 44204 |
| #12 | {OR #8-#11} | 76533 |
| #13 | #4 AND #7 AND #12 in Trials | 41 |

Web of Science, 19-12-2018

| **No.** | **Query** | **Results** |
| --- | --- | --- |
| # 6 | #5 AND #2 AND #1 | 1,305 |
| # 5 | #4 OR #3 | 868,558 |
| # 4 | TOPIC: ("Hepatocyte-Stimulating Factor*" OR "Hybridoma Growth Factor*" OR IL-7 OR IL7 OR Lymphopoietin* OR IL-8 OR IL8 OR "Monocyte-Derived Neutrophil-Activating Peptide*" OR "Anionic Neutrophil-Activating Peptide" OR "Chemokine CXCL8" OR "CXCL8 Chemokine*" OR "Macrophage-Derived Chemotactic Factor*" OR "Neutrophil Chemotactic Factor*" OR "Neutrophil Activation Factor*" OR "Granulocyte Chemotactic Peptide-Interleukin-8" OR "Monocyte-Derived Neutrophil Chemotactic Factor*" OR "Lymphocyte-Derived Neutrophil-Activating Peptide*" OR "Alveolar Macrophage Chemotactic Factor-I" OR AMCF-I OR IL-9 OR IL9 OR "T-Cell Growth Factor P40" OR "P40 T-Cell Growth Factor*" OR IL-10 OR IL10 OR CSIF-10 OR "Cytokine Synthesis Inhibitory Factor*" OR IL-11 OR IL11 OR "Adipogenesis Inhibitory Factor*" OR IL-12 OR IL12 OR "Edodekin Alfa" OR "Natural Killer Cell Stimulatory Factor*" OR "Cytotoxic Lymphocyte Maturation Factor" OR IL-12p35 OR IL12p35 OR IL-12p40 OR IL12p40 OR IL-13 OR IL13 OR IL-15 OR IL15 OR IL-16 OR IL16 OR "Lymphocyte Chemoattractant Factor*" OR "LCF Factor*" OR IL-17* OR IL17* OR CTLA-8 OR CTLA8 OR "Cytotoxic T lymphocyte-Associated Antigen 8" OR IL-18 OR IL18 OR "IFN-gamma-Inducing Factor*" OR IL-23 OR IL23 OR IL-23p19 OR IL-23 p19 OR "IL23 p19" OR IL23p19 OR IL-27 OR IL27 OR Monokine* OR "Tumor Necrosis Factor*" OR TNF OR "TNF Receptor Ligand*" OR TNFalpha OR TNF-alpha OR Cachectin OR "TNF Superfamily") | 375,343 |
| # 3 | TOPIC: (Cytokine* OR Interferon* OR "IFN alpha*" OR Interleukin* OR IL-1 OR IL1 OR "T Helper Factor*" OR "Lymphocyte Activating Factor*" OR "Macrophage Cell Factor*" OR "Epidermal Cell Derived Thymocyte-Activating Factor*" OR Hematopoietin-1 OR Catabolin OR IL-2 OR IL2 OR TCGF OR "Lymphocyte Mitogenic Factor*" OR "T-Cell Growth Factor*" OR "Thymocyte Stimulating Factor*" OR Ro-23-6019 OR Ro236019 OR Ro-236019 OR RU-49637 OR RU49637 OR IL-3 OR IL3 OR "Mast-Cell Colony-Stimulating Factor*" OR "Multipotential Colony-Stimulating Factor*" OR "P-Cell Stimulating Factor*" OR "Erythrocyte Burst-Promoting Factor*" OR Hematopoietin-2 OR IL-4 OR IL4 OR "B-Cell Stimulating Factor*" OR BCGF-1 OR Binetrakin OR BSF-1 OR "Mast Cell Growth Factor-2" OR MCGF-2 OR "B Cell Stimulatory Factor-1" OR "B-Cell Growth Factor*" OR "B-Cell Proliferating Factor*" OR "B-Cell Stimulatory Factor 1" OR IL-5 OR IL5 OR BCGF-II OR "Eosinophil Differentiation Factor*" OR "T-Cell Replacing Factor*" OR "T-Cell-Replacing Factor*" OR "B-Cell Growth Factor-II" OR IL-6 OR IL6 OR "Differentiation Factor 2, B Cell" OR "IFN-beta 2" OR MGI-2 OR "Myeloid Differentiation-Inducing Protein*" OR "Plasmacytoma Growth Factor*" OR "B Cell Stimulatory Factor-2" OR "B-Cell Differentiation Factor" OR "B-Cell Stimulatory Factor-2" OR BSF-2) | 735,896 |
| # 2 | TOPIC: (inflamm* or proinflamm* or antiinflamm*) | 964,281 |
| # 1 | TOPIC: (Sciatic* or slipped dis* or "Intervertebral Disc Displacement*" or "Intervertebral Disk Displacement*" or "herniated dis*" or "prolapsed dis*" or "radicular pain*" or "disc herniation*" or "disk herniation*") | 89,866 |
|  |  |  |

Indexes=SCI-EXPANDED, SSCI, ESCI Timespan=All years

| Database | Voor ontdubbelen | Na ontdubbelen |
| --- | --- | --- |
| PubMed | 980 |  |
| Embase.com | 1435 |  |
| Web of Science | 1305 |  |
| CENTRAL | 41 |  |
| Totaal | 3761 | 2076 |
