# Supplementary material for: Inflammatory biomarkers in patients with sciatica: a systematic review
Source: BMC Musculoskelet Disord. 2019 Apr 9;20:156. doi: 10.1186/s12891-019-2541-0 (PMC6456959; doi:10.1186/s12891-019-2541-0)
Supplement: Supplementary file 3 — The Quality in Prognosis Studies Tool (QUIPS). (DOCX 17 kb) [file 12891_2019_2541_MOESM3_ESM.docx]

**Additional file 3**

The adjusted Quips tool for bias assessment

| Domains of the QUIPS risk of bias assessment. | | | | | | | |
| --- | --- | --- | --- | --- | --- | --- | --- |
| Variable | 1. Study Participation | 2. Study Attrition | 3. Prognostic Factor  Measurement | 4. Outcome  Measurement | 5. Study Confounding | 6. Statistical Analysis and Reporting |  |
| Optimal study or characteristics of unbiased study. | The study sample adequately represents the population of interest. | The study data available (i.e., participants not lost to follow-up) adequately represent the study sample. | The PF is measured in a similar way for all participants. | The outcome of interest is measured in a similar way for all participants. | Important potential confounding factors are appropriately accounted for. | The statistical analysis is appropriate, and all primary outcomes are reported. |  |
| Prompting items and considerations. | a. Adequate participation in the study by eligible persons. | a. Adequate response rate for study participants. | a. A clear definition or description of the PF is provided | a. A clear definition of the outcome is provided. | a. All important confounders are measured. | a. Sufficient presentation of data to assess the adequacy of the analytic strategy. |  |
|  | b. Description of the source population or population of interest. | b. Description of attempts to collect information on participants who dropped out. | b. Method of PF measurement is adequately valid and reliable | b. Method of outcome measurement used is adequately valid and reliable. | b. Clear definitions of the important confounders measured are provided. | b. Strategy for model building is appropriate and is based on a conceptual framework or model. |  |
|  | c. Description of the baseline study sample. | c. Reasons for loss to follow-up are provided. | c. Continuous variables are reported or appropriate cut points are used. | c. The method and setting of outcome measurement is the same for all study participants. | c. Measurement of all important confounders is adequately valid and reliable. | c. The selected statistical model is adequate for the design of the study. |  |
|  | d. Adequate description of the sampling frame and recruitment. | d. Adequate description of participants lost to follow-up. | d. The method and setting of measurement of PF is the same for all study participants. |  | d. The method and setting of confounding measurement are the same for all study participants. | d. There is no selective reporting of results. |  |
|  | e. Adequate description of the period and place of recruitment. | e. There are no important differences between participants who completed the study and those who did not. | e. Adequate proportion of the study sample has complete data for the PF. |  | e. Appropriate methods are used if imputation is used for missing confounder data. |  |  |
|  | f. Adequate description of inclusion and exclusion criteria. |  | f. Appropriate methods of imputation are used for missing PF data. |  | f. Important potential confounders are accounted for in the study design. |  |  |
|  |  |  |  |  | g. Important potential confounders are accounted for in the analysis. |  |  |
